# Associations of Alcohol Consumption and Smoking With Disease Risk and Neurodegeneration in Individuals With Multiple Sclerosis in the United Kingdom

**DOI:** 10.1001/jamanetworkopen.2022.0902

**Published:** 2022-03-03

**Authors:** Iris Kleerekooper, Sharon Chua, Paul J. Foster, S. Anand Trip, Gordon T. Plant, Axel Petzold, Praveen Patel

**Affiliations:** 1 Queen Square MS Centre, Department of Neuroinflammation, UCL (University College London) Institute of Neurology, London, United Kingdom; 2 Department of Neuro-ophthalmology, Moorfields Eye Hospital, London, United Kingdom; 3 NIHR (National Institute for Health Research) Biomedical Research Centre, Moorfields Eye Hospital, NHS (National Health Service) Foundation Trust and UCL Institute of Ophthalmology, London, United Kingdom; 4 Dutch Expertise Centre for Neuro-ophthalmology and MS (Multiple Sclerosis) Centre, Departments of Neurology and Ophthalmology, Amsterdam University Medical College, Amsterdam, the Netherlands

## Abstract

**Importance:**

Understanding the effects of modifiable risk factors on risk for multiple sclerosis (MS) and associated neurodegeneration is important to guide clinical counseling.

**Objective:**

To investigate associations of alcohol use, smoking, and obesity with odds of MS diagnosis and macular ganglion cell layer and inner plexiform layer (mGCIPL) thickness.

**Design, Setting, and Participants:**

This cross-sectional study analyzed data from the community-based UK Biobank study on health behaviors and retinal thickness (measured by optical coherence tomography in both eyes) in individuals aged 40 to 69 years examined from December 1, 2009, to December 31, 2010. Risk factors were identified with multivariable logistic regression analyses. To adjust for intereye correlations, multivariable generalized estimating equations were used to explore associations of alcohol use and smoking with mGCIPL thickness. Finally, interaction models explored whether the correlations of alcohol and smoking with mGCIPL thickness differed for individuals with MS. Data were analyzed from February 1 to July 1, 2021.

**Exposures:**

Smoking status (never, previous, or current), alcohol intake (never or special occasions only [low], once per month to ≤4 times per week [moderate], or daily/almost daily [high]), and body mass index.

**Main Outcomes and Measures:**

Multiple sclerosis case status and mGCIPL thickness.

**Results:**

A total of 71 981 individuals (38 685 women [53.7%] and 33 296 men [46.3%]; mean [SD] age, 56.7 [8.0] years) were included in the analysis (20 065 healthy control individuals, 51 737 control individuals with comorbidities, and 179 individuals with MS). Modifiable risk factors significantly associated with MS case status were current smoking (odds ratio [OR], 3.05 [95% CI, 1.95-4.64]), moderate alcohol intake (OR, 0.62 [95% CI, 0.43-0.91]), and obesity (OR, 1.72 [95% CI, 1.15-2.56]) compared with healthy control individuals. Compared with the control individuals with comorbidities, only smoking was associated with case status (OR, 2.30 [95% CI, 1.48-3.51]). High alcohol intake was associated with a thinner mGCIPL in individuals with MS (adjusted β = −3.09 [95% CI, −5.70 to −0.48] μm; *P* = .02). In the alcohol interaction model, high alcohol intake was associated with thinner mGCIPL in control individuals (β = −0.93 [95% CI, −1.07 to −0.79] μm; *P* < .001), but there was no statistically significant association in individuals with MS (β = −2.27 [95% CI, −4.76 to 0.22] μm; *P* = .07). Smoking was not associated with mGCIPL thickness in MS. However, smoking was associated with greater mGCIPL thickness in control individuals (β = 0.89 [95% CI, 0.74-1.05 μm]; *P* < .001).

**Conclusions and Relevance:**

These findings suggest that high alcohol intake was associated with retinal features indicative of more severe neurodegeneration, whereas smoking was associated with higher odds of being diagnosed with MS.

## Key Points

**Question:**

How are modifiable risk factors such as alcohol consumption, smoking, and obesity associated with disease risk and neurodegeneration in individuals with multiple sclerosis (MS)?

**Findings:**

This cross-sectional study of 71 981 individuals in the United Kingdom found that high alcohol consumption was associated with retinal features indicative of more severe neurodegeneration, whereas smoking was associated with higher odds of being diagnosed with MS.

**Meaning:**

These findings suggest that current recommendations for the general population regarding smoking and moderating alcohol consumption may be particularly relevant for individuals who have been diagnosed with MS or who are at risk for the disease.

## Teaser

This cross-sectional study investigates the associations of alcohol consumption and smoking with a diagnosis of multiple sclerosis and thickness of the macular ganglion cell layer and inner plexiform layer in a community-based sample from the UK Biobank study.

## Introduction

Multiple sclerosis (MS) is an immune-mediated, demyelinating disorder of the central nervous system, predominantly affecting women of child-bearing age.^1^ Both genetic and environmental factors are known to play important roles in the pathophysiology of MS.^1,2,3^ Understanding the role of modifiable risk factors, such as smoking, alcohol intake, and obesity, is important to guide clinical counseling.^4,5^ Smoking is known to increase the risk of developing MS,^6^ and advising patients to stop smoking to reduce the risk of conversion from clinically isolated syndrome (CIS) to MS is an important part of patient guidance.^7^ Less is known about how health behaviors influence neurodegeneration in MS. Brain atrophy occurs in MS from disease diagnosis,^8^ but its underlying mechanisms remain poorly understood. Reduced brain volumes are associated with more severe disability,^9^ particularly in the cognitive domains.^10,11^ Ameliorating neurodegeneration has therefore become an important treatment goal.^12^

Studies investigating brain atrophy are hampered by long magnetic resonance imaging scanning protocols. Retinal thickness measures have been identified as a surrogate for brain volume,^13,14^ because the retina is developmentally and anatomically part of the central nervous system but is more easily assessable for imaging. Optical coherence tomography (OCT) provides fast, noninvasive retinal imaging.^15^ Thickness of the macular ganglion cell and inner plexiform layer (mGCIPL) correlates with brain volume measures in the general population^16^ and in patients with MS.^14^ Retinal atrophy, independent of damage inflicted by optic neuritis, occurs from the early stages of disease^15,17^ and correlates with MS disability scores.^18,19^

Because smoking and alcohol consumption are correlated behaviors,^20,21^ it is difficult to disentangle their respective effects. In the general population, smoking and alcohol intake separately have been found to be related to brain atrophy,^22,23,24,25,26,27,28,29,30^ and daily alcohol intake and maternal smoking during pregnancy have also been found to be associated with thinner retinal thickness.^31,32^

Smoking may also increase disease activity, disability progression,^6^ and neurodegeneration^5^ in MS. However, the findings of studies investigating associations between smoking and brain volume in MS have been inconclusive^5,33,34^ and were not adjusted for the potential confounding effects of alcohol use. How alcohol affects MS is less clear, with both protective^35,36^ and adverse effects^4,37^ reported, and to our knowledge the association between alcohol consumption and brain atrophy in MS has not yet been investigated. Correlations of alcohol intake with health outcomes are complex, frequently following a J-shaped curve. Moderate alcohol consumption may be protective against developing cardiac infarction^38^ and cataract,^39^ for example. However, with regard to brain volume^27,30^ and mortality,^38^ most evidence points to a linear negative correlation with alcohol, without a protective effect of moderate consumption.

The purpose of this study was to investigate the associations of alcohol consumption and smoking with an MS diagnosis and mGCIPL thickness in a community-based cohort study comprising more than 70 000 adults in the United Kingdom. A better understanding of the roles of these modifiable risk factors may lead to better health outcomes and quality of life in MS.

## Methods

### Participants

The participants of this cross-sectional study were adults aged 40 to 69 years who were registered in the National Health Service (NHS) and who participated in an expanded ophthalmic protocol, including OCT, of the UK Biobank (UKBB) baseline visit between January 1, 2009, and December 31, 2010.^40^ The North West Multi-Centre Research Ethics Committee approved the UKBB study protocol in accordance with the tenets of the Declaration of Helsinki. All participants gave written informed consent. This study followed the Strengthening the Reporting of Observational Studies in Epidemiology (STROBE) reporting guideline.

Participants were categorized as having MS, no comorbidities, or comorbidities based on clinician-controlled *International Statistical Classification of Diseases and Related Health Problems, Tenth Revision* (*ICD-10*), disease codes, available through record linking with the NHS.^41^ The *ICD-10* code for MS, G35, is applicable to all forms of MS diagnosed by clinicians within the NHS, but does not specify which tests were used to reach a diagnosis. The cumulative disease burden was calculated by counting the number of *ICD-10* codes per participant. Individuals without MS were categorized as healthy if this cumulative disease burden was 0 and having comorbidities if it was 1 or greater.

### Data Collection

In brief, undilated macular spectral domain OCT scans of both eyes were obtained in a dark room with a 3-dimensional scanner (3D OCT-1000 Mk-II; Topcon Corporation), in line with the Advised Protocol for OCT Study Terminology and Elements guidelines^42,43^ as described in detail previously.^44^ An extensive quality-control protocol, combining both automated and manual checks, was used to ensure sufficient image quality, complying with OSCAR-IB criteria (obvious problems; sufficient signal; correct centering of ring scan; algorithm failure; visible retinal pathology; well-illuminated fundus; and central measurement beam)^45^ and accurate layer segmentation.^32,44^

Participants completed a self-administered touch-screen questionnaire on health-related behaviors, demographics, and socioeconomic data. Smoking and alcohol status were determined by asking the questions “Do you smoke tobacco?” (never, previously, or currently) and “How often do you drink alcohol?” (never, special occasions only, 1-3 times per month, 1-2 times per week, 3-4 times per week, daily, or almost daily). Responses to the alcohol-related question were consolidated into the categories low (never or special occasions only), moderate (drinking once per month to ≤4 times per week), and high (daily or almost daily). A sensitivity analysis was performed with the original alcohol intake levels and was reported if these results were materially different. Household passive smoking was determined by asking the question “Do any household members smoke tobacco?”

Race and ethnicity responses were collected given their know association with MS risk and were placed into 5 categories: (1) Asian (including Asian or Asian British, Bangladeshi, Chinese, Indian, Pakistani, or other Asian background), (2) Black (including African, Black or Black British, Caribbean, or other Black background), (3) White (including British, Irish, White, or other White background), (4) other and/or multiracial (White and Asian, White and Black African, White and Black Caribbean, other mixed background, mixed, or other ethnic group), and (5) missing (unknown and missing). Corneal compensated intraocular pressure (IOP) measurements were performed with an ocular response analyzer (Reichert Ophthalmic Instruments) from 1 eye. Weight and height were measured by trained trial personnel with a body composition analyzer (BV-418 MA; Tanita) and a medical measuring rod (Seca 202; Seca), respectively. Body mass index (BMI) was calculated as weight in kilograms divided by the height in meters squared and transformed into a categorical variable (<18.0 indicates underweight; 18.0-24.9, healthy weight; 25.0-30.0, overweight; and >30.0, obesity). Postal codes were used to determine Townsend Deprivation Score,^46^ which was transformed into a categorical variable based on quartiles of equal group size.

### Statistical Analysis

Data were analyzed from February 1 to July 1, 2021. Data distributions were tabulated using summary statistics for continuous variables (mean [SD]) and cross-tabulations (including percentages) for categorical variables. Data were inspected visually for normality and inconsistencies. Missing data were tabulated and were excluded from analysis.

#### MS Case Status

Univariable followed by multivariable logistic regression was used to identify factors associated with the odds of having an MS diagnosis. Results were reported with odds ratios (ORs) and 95% CIs. The healthy control group and the control group with comorbidities were used separately. Covariates considered were age, sex, Townsend deprivation score, race and ethnicity, and BMI. The assumed associations among the exposures, potential confounders, and outcomes are presented in a directed acyclic graph in the eFigure in Supplement 1.

#### mGCIPL Thickness in MS

Subsequently, we built a multivariable model investigating the associations of alcohol use and smoking with mGCIPL thickness in individuals with MS. To account for intereye correlations, generalized estimating equations were used.^42,43^ A sensitivity analysis was performed with linear regression on the mean mGCIPL value of both eyes. Spearman correlations were performed between all covariates to identify evidence for multicollinearity (ρ > 0.5). Potential confounding factors were explored through univariable associations of covariates with exposures (smoking and alcohol intake) and outcome (mGCIPL thickness), separately. This was tested using χ^2^ tests for categorical variables and Kruskal-Wallis tests for continuous variables. Age, sex, and IOP were included in the final model a priori. To explore potential multiplicative effects of combined smoking and alcohol use, we tested a model including interaction terms for the alcohol and smoking variables.

#### Effect Modification in MS

Finally, we explored whether associations of smoking and alcohol use with mGCIPL thickness differed between control individuals and individuals with MS. Two generalized estimating equations models were run on the complete cohort, including variables for MS case status, alcohol intake, and smoking status as well as an interaction term for MS and either smoking status or alcohol intake. These models were inspected for significant (*P* < .05) results for the Wald test of the interaction terms.

#### Analysis

A statistical significance threshold of 2-sided *P* < .05 was used. R software, version 4.1.1, and R Studio, version 1.4.1103 (R Project for Statistical Computing) were used for all statistical analyses.

## Results

### Participants

**Figure 1.**
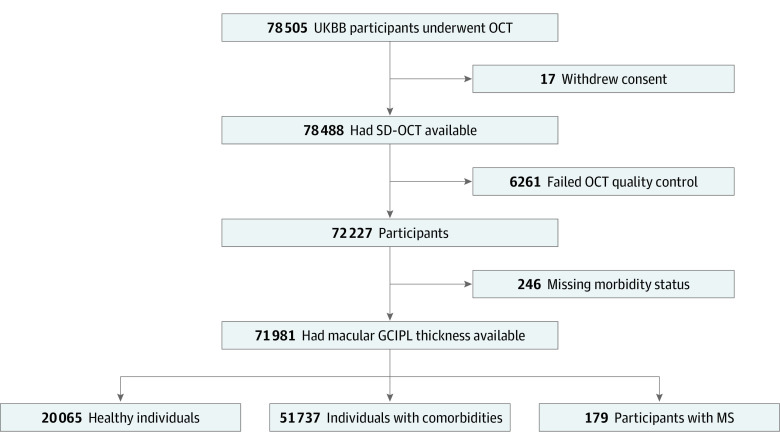
Study Flowchart Individuals with multiple sclerosis (MS) were defined as those with an *International Statistical Classification of Diseases and Related Health Problems, Tenth Revision* (*ICD-10*), disease code of G35, indicating clinician-diagnosed MS. Healthy control individuals were those who had 0 *ICD-10* disease codes registered, whereas control individuals with comorbidities had 1 or more (non-MS) *ICD-10* disease codes registered. GCIPL indicates ganglion cell and inner plexiform layer; SD-OCT, spectral domain optical coherence tomography; and UKBB, UK Biobank study.

A total of 71 981 participants were included in the study (38 685 women [53.7%] and 33 296 men [46.3%]; mean [SD] age, 56.7 [8.0] years). In terms of diagnoses, 179 individuals with MS, 20 065 healthy control individuals and 51 737 control individuals with comorbidities were included (Figure 1). Among these participants, 130 individuals with MS (72.6%) and 38 555 control individuals (53.6%) were women; 49 individuals with MS (27.4%) and 33 247 control individuals (46.3%) were men (Table 1).^1^ Individuals with MS had a mean (SD) age of 55.6 (7.7) years; control individuals, 56.6 (7.9) years. Three individuals with MS (1.7%) were classified as having underweight BMI, and this level was collapsed with healthy weight.

**Table 1.** Study Cohort Characteristics

Characteristic	Participant group^a^		
	Healthy control individuals (n = 20 065)	Control individuals with comorbidities (n = 51 737)	Individuals with MS (n = 179)
Sex			
Women	10 064 (50.2)	28 491 (55.1)	130 (72.6)
Men	10 001 (49.8)	23 246 (44.9)	49 (27.4)
Age, mean (SD), y	55.2 (7.9)	57.2 (8.0)	55.6 (7.7)
Smoking status			
Never	12 071 (60.2)	27 896 (53.9)	81 (45.3)
Previous	6210 (30.9)	18 666 (36.1)	63 (35.2)
Current	1702 (8.5)	4858 (9.4)	33 (18.4)
Missing/no answer	82 (0.4)	317 (0.6)	2 (1.1)
Alcohol consumption^b^			
Low	3267 (16.3)	10 832 (20.9)	47 (26.3)
Moderate	12 228 (60.9)	30 251 (58.5)	89 (49.7)
High	4515 (22.5)	10 489 (20.3)	41 (22.9)
Missing or no answer	55 (0.3)	165 (0.3)	2 (1.1)
Race and ethnicity			
Asian	705 (3.5)	1594 (3.1)	4 (2.2)
Black	537 (26.8)	1394 (26.9)	3 (1.7)
White	18 232 (90.9)	47 333 (91.5)	168 (93.9)
Other or mixed^c^	529 (2.6)	1205 (2.3)	2 (1.1)
Missing or no answer	62 (0.3)	211 (0.4)	2 (1.1)
Townsend deprivation index, quartile			
Lowest	5234 (26.1)	12 720 (24.6)	33 (18.4)
Low-middle	4975 (24.8)	12 958 (25.0)	46 (25.7)
High-middle	5063 (25.2)	12 865 (24.9)	43 (24.0)
Highest	4770 (23.8)	13 135 (25.4)	57 (31.8)
Missing or no answer	23 (0.1)	59 (0.1)	0
BMI group			
Low or normal weight (<18.0-24.9)	7841 (39.1)	16 731 (32.3)	60 (33.5)
Overweight (25.0-30.0)	8549 (42.6)	21 767 (42.1)	67 (37.4)
Obese (>30.0)	3603 (18.0)	13 026 (25.2)	43 (24.0)
Missing	72 (0.4)	213 (0.4)	9 (5.0)
Intraocular pressure			
Mean (SD), mm Hg	16.0 (4.3)	15.9 (4.3)	15.1 (3.7)
Missing	657 (3.3)	1618 (3.1)	12 (6.7)
Household passive smoking			
Yes	1820 (9.1)	4896 (9.5)	21 (11.7)
No	16 979 (84.6)	42 926 (83.0)	134 (74.9)
Missing	1266 (6.3)	3915 (7.6)	24 (13.4)
mGCIPL, mean (SD), μm			
Thickness	72.8 (6.0)	72.2 (6.0)	67.8 (6.3)
IEPD	2.6 (3.4)	2.8 (3.6)	6.4 (6.3)
IEAD	2.0 (2.5)	2.1 (2.7)	4.5 (4.7)

Abbreviations: BMI, body mass index (calculated as weight in kilograms divided by height in meters squared); IEAD, intereye absolute difference in mGCIPL; IEPD, intereye percent difference in mGCIPL; mGCIPL, macular ganglion cell layer and inner plexiform layer; MS, multiple sclerosis.

^a^ Unless otherwise indicated, data are expressed as number (%) of participants. Percentages have been rounded and may not total 100.

^b^ Defined as never or special occasions only (low), once per month to no more than 4 times per week (moderate), or daily or almost daily (high).

^c^ Includes White and Asian, White and Black African, White and Black Caribbean, other mixed background, mixed, or other ethnic group.

### Odds of MS Case Status

**Figure 2.**
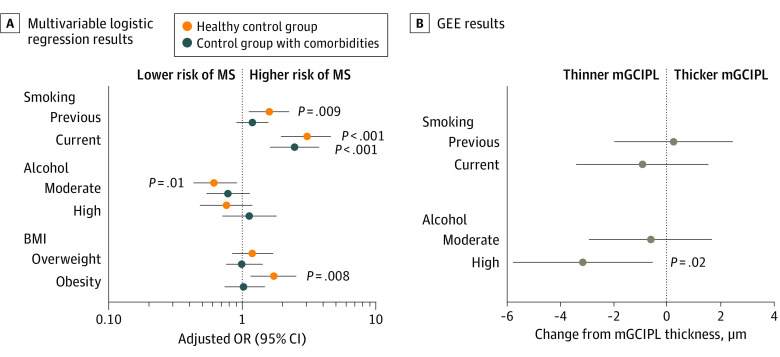
Line Plots Visualizing Associations of Modifiable Risk Factors With Multiple Sclerosis (MS) Risk and Macular Ganglion Cell and Inner Plexiform Layer (mGCIPL) Thickness A, Multivariable logistic regression results visualizing factors associated with risk of being diagnosed with MS. Circles represent odds ratios; whiskers represent 95% CIs. Analysis is adjusted for age, sex, and Townsend deprivation score. The x-axis is log-transformed. B, Multivariable generalized estimating equations (GEEs) results visualizing the associations with smoking status and alcohol intake (adjusted for age, sex, Townsend deprivation score, and intraocular pressure [IOP]) with mGCIPL thickness in individuals with MS. Reference groups include individuals who never smoked, had low alcohol use, and had a healthy body mass index (BMI; calculated as weight in kilograms divided by height in meters squared). Alcohol consumption was classified as never or special occasions only (low), once per month to no more than 4 times per week (moderate), or daily or almost daily (high); BMI was classified as underweight or healthy weight (<18.0 to 24.9), overweight (25.0-30.0), and obesity (>30.0). OR indicates odds ratio.

Univariable logistic analysis and subsequent multivariable logistic regression (Figure 2A and eTable 1 in Supplement 1) were performed using the healthy control group and the control group with comorbidities separately. Ethnicity was not included because only 9 individuals with MS (5.0%) were of an ethnicity other than White. Household passive smoking was not associated with the odds of MS (OR for healthy control group, 1.46 [95% CI, 0.90-2.27]; OR for control group with comorbidities, 1.37 [95% CI, 0.84-2.13]) (eTable 1 in Supplement 1), and this variable was not included in multivariable analysis.

Compared with never having smoked, being a current or previous smoker was associated with ORs of 3.05 (95% CI, 1.95-4.64) and 1.59 (95% CI, 1.12-2.25), respectively, for having MS case status when using a healthy control group and ORs of 2.30 (95% CI, 1.48-3.51) and 1.25 (95% CI, 0.88-1.77), respectively, for having MS case status when using a control group with comorbidities. Moderate alcohol intake was associated with lower odds of MS case status using the healthy control group (adjusted OR [aOR], 0.62 [95% CI, 0.43-0.91]) but not the control group with comorbidities (aOR, 0.81 [95% CI, 0.56-1.18]). Having obesity was significantly associated with an increased odds of MS when comparing the healthy control group (aOR, 1.72 [95% CI, 1.15-2.56]) but not the control group with comorbidities (aOR, 1.02 [95% CI, 0.68-1.51]).

### mGCIPL Thickness in MS

There was a significant univariable negative association of mGCIPL thickness with high alcohol consumption (β = −2.94 [95% CI, −5.49 to −0.68] μm; *P* = .02) (Table 2). Current smoking (β = −1.26 [95% CI, −4.14 to 0.93] μm; *P* = .30), previous smoking (β = −0.34 [95% CI, −2.36 to 2.02] μm; *P* = .75), and household passive smoking (β = −0.76 [95% CI, −2.43 to 2.01] μm; *P* = .61) were not significantly associated with mGCIPL thickness. Similarly, there were no associations of overweight BMI (β = −0.41 [95% CI, −3.34 to 2.63] μm; *P* = .72]) or obesity BMI (β = −0.41 [95% CI, −4.58 to 1.67] μm; *P* = .77]) with mGCIPL thickness.

**Table 2.** Univariable and Multivariable GEEs Investigating Associations With mGCIPL Thickness in MS Among 164 Individuals

Characteristic	Univariable analysis^a^				Multivariable analysis^b^		
	β (95% CI)	*P* value	*P* value for trend	No. of observations	Adjusted β (95% CI)	*P* value	*P* value for trend
Sex							
Women	NA	NA	NA	179	NA	NA	NA
Men	−1.37 (−1.12 to 3.26)	.20			−1.02 (−1.22 to 3.25)	.37	
Age per unit increase	0.00 (−0.15 to 0.10)	.99	NA	179	−0.04 (−0.17 to 0.07)	.36	NA
Smoking status							
Never	NA	NA	.33	177	NA	NA	NA
Previous	−0.34 (−2.36 to 2.02)	.75			0.37 (−1.83 to 2.57)	.74	
Current	−1.26 (−4.14 to 0.93)	.30			−0.66 (−3.21 to 1.89)	.61	
Alcohol consumption^c^							
Low	NA	NA	.02	177	NA	NA	.02
Moderate	−0.15 (−2.62 to 2.05)	.90			−0.56 (−2.82 to 1.71)	.63	
High	−2.94 (−5.49 to −0.68)	.02			−3.09 (−5.70 to −0.48)	.02	
Townsend deprivation index^d^							
Lowest quartile	NA	NA	.88	179	NA	NA	NA
Low-middle quartile	−0.91 (−3.34 to 2.63)	.54			−0.32 (−3.33 to 2.69)	.84	
High-middle quartile	−1.34 (−4.58 to 1.67)	.39			−1.45 (−4.70 to 1.79)	.38	
Highest quartile	−0.34 (−3.15 to 2.85)	.82			−0.30 (−343 to 2.83)	.85	
BMI group							
Low/normal weight (<18.0 to 24.9)	NA	NA	NA	170	NA	NA	NA
Overweight (25.0-30.0)	−0.41 (−3.34 to 2.63)	.72			NA	NA	
Obesity (>30.0)	−0.41 (−4.58 to 1.67)	.77			NA	NA	
IOP per unit increase	1.61 (−3.15 to 2.85)	.29	NA	167	0.21 (−0.09 to 0.51)	.17	NA
Household passive smoking							
No	NA	NA	NA	156	NA	NA	NA
Yes	−0.76 (−2.43 to 2.01)	.61			NA	NA	NA

Abbreviations: BMI, body mass index (calculated as weight in kilograms divided by height in meters squared); GEEs, generalized estimating equations; IOP, intraocular pressure; mGCIPL, macular ganglion cell and inner plexiform layer; MS, multiple sclerosis; NA, not applicable.

^a^ Investigates associations with explanatory variables (alcohol consumption and smoking status) and other covariates with mGCIPL thickness in individuals diagnosed with MS.

^b^ Investigates the associations between alcohol consumption and smoking status with mGCIPL thickness in individuals diagnosed with MS, adjusted for confounders.

^c^ Defined as never or special occasions only (low), drinking once per month to no more than 4 times per week (moderate), or daily or almost daily (high).

^d^ Scores range from −6.3 to 9.4, with higher scores indicating higher levels of deprivation.

Within the MS cohort, there was a significant association of smoking with sex (*P* = .01). There was no association with Townsend deprivation index (*P* = .07) and of alcohol intake with sex (*P* = .06) and with IOP (*P* = .05). Because BMI was not associated with mGCIPL or either explanatory variable, it was not taken forward for multivariable analysis. To explore potential multiplicative effects of combined alcohol intake and smoking, a model with interaction terms was created (eTable 2 in Supplement 1), which identified no evidence of interaction.

The final multivariable generalized estimating equation model, adjusted for smoking status, sex, age, Townsend deprivation score, and IOP, identified a significant association of high alcohol consumption with a thinner mGCIPL (adjusted β = −3.09 [95% CI, −5.70 to −0.48] μm; *P* = .02) (Table 2 and Figure 2B). There was a significant linear trend association across the alcohol intake parameters (adjusted β = −1.55 [95% CI, −3.20 to −0.35] μm; *P* = .02). No significant associations were identified for previous smokers or current smokers, compared with those who never smoked. A sensitivity analysis using multivariable linear regression found similar results for high alcohol intake (adjusted β = −3.12 [95% CI, −5.55 to −0.46] μm; *P* = .04). Finally, after excluding the 9 individuals with MS who were not of White ethnicity, high alcohol intake remained significantly associated with mGCIPL (adjusted β = −3.01 [95% CI, −5.41 to −0.44] μm; *P* = .03).

### Differences in Associations Based on MS Case Status

**Figure 3.**
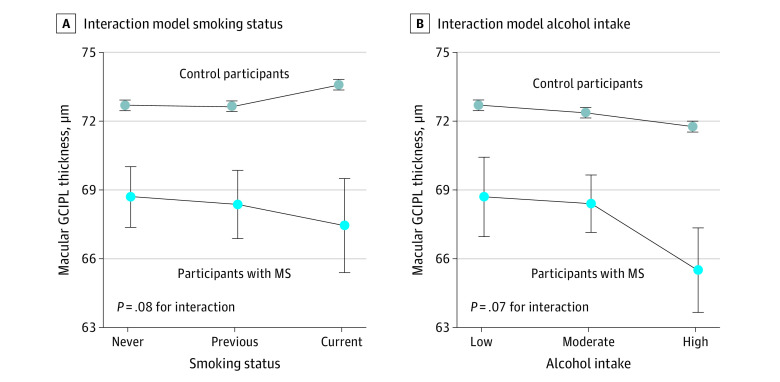
Differences in Associations With Macular Ganglion Cell and Inner Plexiform Layer (mGCIPL) Thickness Based on Multiple Sclerosis (MS) Diagnosis Status Modeled estimates of the associations of mGCIPL with alcohol consumption and smoking status for the entire cohort, plotted for individuals with MS and control individuals separately. A, Interaction model including interaction term for smoking status and MS diagnosis. B, Interaction model including interaction term for alcohol intake and MS diagnosis. Whiskers represent 95% CIs.

The smoking interaction model suggested that the association of smoking with mGCIPL thickness differed by MS case status, because current smoking was associated with thicker mGCIPL in the control group (β = 0.89 [95% CI, 0.74-1.05] μm; *P* < .001), but was not associated with mGCIPL in individuals with MS (β = −2.14 [95% CI, −4.52 to 0.23] μm; *P* = .08) (eTable 3 in Supplement 1). In the alcohol interaction model, high alcohol intake was associated with thinner mGCIPL in control individuals (β = −0.93 [95% CI, −1.07 to −0.79] μm; *P* < .001), but there was no statistically significant association in MS (β = −2.27 [95% CI, −4.76 to 0.22] μm; *P* = .07) (Figure 3).

## Discussion

In this cross-sectional study, smoking was associated with an increased odds of having an MS diagnosis and high alcohol consumption was associated with a thinner mGCIPL in individuals with MS. Paradoxically, moderate alcohol intake was associated with a lower odds of having been diagnosed with MS. The associations of smoking and alcohol use with mGCIPL thickness may be different in individuals with MS compared with control individuals.

Our findings replicate associations of smoking and obesity with increased odds of MS diagnosis status.^1,6,47^ Only smoking remained significantly associated with odds of having an MS diagnosis when using the control group with comorbidities. This finding illustrates the importance of choice of control group, because risk factors and protective factors are likely similar across various diseases, which can obscure associations.

Moderate alcohol consumption was associated with a lower odds of having an MS diagnosis. A protective effect of moderate alcohol consumption on MS risk has been reported before,^35,36^ although an increased risk has been reported as well.^4,37^ Owing to the cross-sectional nature of the present study, the “sick quitters effect”—that is, the tendency to quit or profoundly limit alcohol intake when ill could have influenced our findings.^48^

This study identified a novel association of high alcohol consumption with a thinner mGCIPL in MS, whereas there was no association with smoking. Participants with self-reported high alcohol intake had almost 5% thinner mGCIPLs, a substantial difference in retinal thickness. This seems to be in line with reported associations of alcohol use with lower brain volumes^27,30^ and a large recent population-based study,^30^ which found that alcohol consumption explained 7.7% of variance in gray matter volume, compared with 1.7% by smoking status. A previous study^34^ found that smoking was not associated with retinal thickness in MS. Other studies^5^ identified significant correlations of smoking with brain atrophy in MS, but these studies were not adjusted for alcohol consumption. We observed a linear negative association of alcohol with mGCIPL thickness in MS, without protective effects of moderate alcohol consumption. This resembles previous findings in the general population.^27,30^

The observed association of high alcohol consumption with lower mGCIPL thickness in MS remains an imperfect surrogate for clinically relevant metrics such as disability load. Smoking has been shown to be associated with increased disease severity.^6^ In contrast, alcohol has been reported to ameliorate MS disease severity and progression,^36,49^ particularly in smokers,^50^ while at the same time being associated with increased cerebral lesion load.^51^ However, as in the present study, these studies were cross-sectional, and the sick quitters effect could have influenced the results. The association of alcohol consumption with MS severity is complex, and future prospective studies using measures of physical and cognitive disability are needed to elucidate these questions. Importantly, the present study does not provide evidence of a health benefit for patients with MS to refrain from alcohol consumption.

The data reported in this study suggest that associations of both smoking and alcohol intake with mGCIPL thickness may be different for individuals with MS compared with control individuals, with individuals diagnosed with MS appearing to be more susceptible to the neurodegenerative effects of these adverse health behaviors. Although these findings did not reach statistical significance and need to be interpreted with caution, these may still represent clinically important processes, given their large effect sizes. These findings will need to be replicated in an independent cohort to be confirmed. Our findings suggest that neurodegenerative processes occurring in MS may interact with neurotoxic effects of alcohol and smoking, resulting in greater neuronal cell death and axonal loss. Ethanol and its metabolite acetaldehyde are directly neurotoxic.^52,53^ In addition, both alcohol and smoking are related to microvascular dysfunction and oxidative stress,^54,55^ which could aggravate MS pathophysiological processes, because mitochondrial failure may play an important role.^56,57^ However, the association of thinner mGCIPL with high alcohol intake could also be related to retinal mechanisms instead of neuroaxonal loss. For example, the high energy use of the retina may make it sensitive to damage due to alcohol-related increases in oxidative stress,^58^ and higher alcohol intake has been found to be associated with increased risk of glaucoma.^59^ This might have influenced our results, although our analysis was adjusted for IOP.

### Strengths and Limitations

The strengths of this study include the large community-based data set that provided extensive and high-quality data. This study also has some limitations. Although the questionnaire on exposure status has not been formally validated, previous studies^38,39^ demonstrated good performance. However, the self-reported nature of exposure status classification could have caused misclassification. This would have most likely been nondifferential misclassification, biasing effect estimates to zero, although individuals with MS may have had different likelihoods of overestimating and underestimating their health behaviors compared with healthy control individuals. Furthermore, alcohol consumption in this study was not determined with regard to quantity of alcohol units consumed, because this information was missing for a large proportion. This means we were not able to quantify alcohol intake more precisely, identify binge-drinking behaviors, or distinguish the effects of various alcoholic beverage types, which might have shown distinctive health effects of red wine, as was shown before in relation to cataract.^39^ A further limitation was the low response rate of the UKBB, with an underrepresentation of individuals who belong to ethnic minority groups or have a lower socioeconomic status. In particular, individuals with MS who participated in the UKBB may not have been representative of the general population with MS because they were aged 40 to 69 years and may have had milder disease, enabling them to travel to study centers. In addition, we did not have sufficiently reliable information on optic neuritis status, disease duration, or disability to take these factors into account, which could have caused residual confounding.

## Conclusions

This cross-sectional study found that high alcohol consumption was associated with more pronounced retinal features of neurodegeneration, although moderate alcohol consumption was associated with lower odds of being diagnosed with MS. Smoking was associated with increased odds of having an MS diagnosis. Further research is necessary to confirm the results of this study, in particular the complex associations of alcohol consumption with MS severity. The presented findings suggest that current recommendations for the general population regarding smoking and moderating alcohol consumption may be particularly relevant for individuals who have been diagnosed with MS or who are at risk for the disease.

## Notes

Supplement 1. **eTable 1.** Univariable and Multivariable Logistic Regression—Odds of Multiple Sclerosis Case Status **eTable 2.** Testing for Multiplicative Interactions of Alcohol and Smoking in Multiple Sclerosis **eTable 3.** Effect Modification of Alcohol and Smoking Effects by Multiple Sclerosis Diagnosis **eFigure.** Directed Acyclic Graph (DAG) Visualizing the Assumed Associations Among the Exposures, Outcomes and Potential Confounders in This Study

Supplement 2.
Nonauthor Collaborators
