# Supplementary material for: Associations of Alcohol Consumption and Smoking With Disease Risk and Neurodegeneration in Individuals With Multiple Sclerosis in the United Kingdom
Source: JAMA Netw Open. 2022 Mar 3;5(3):e220902. doi: 10.1001/jamanetworkopen.2022.0902 (PMC8895260; doi:10.1001/jamanetworkopen.2022.0902)
Supplement: Supplement 1. — eTable 1. Univariable and Multivariable Logistic Regression—Odds of Multiple Sclerosis Case Status eTable 2. Testing for Multiplicative Interactions of Alcohol and Smoking in Multiple Sclerosis eTable 3. Effect Modification of Alcohol and Smoking Effects by Multiple Sclerosis Diagnosis eFigure. Directed Acyclic Graph (DAG) Visualizing the Assumed Associations Among the Exposures, Outcomes and Potential Confounders in This Study [file jamanetwopen-e220902-s001.pdf]

Supplementary Online Content

Kleerekooper I, Chua S, Foster PJ, et al; UK Biobank Eye and Vision Consortium. Associations of alcohol consumption and smoking with disease risk and neurodegeneration in individuals with multiple sclerosis in the United Kingdom. *JAMA Netw Open*. 2022;5(3):e220902. doi:10.1001/jamanetworkopen.2022.0902

eTable 1. Univariable and Multivariable Logistic Regression—Odds of Multiple Sclerosis Case Status

eTable 2. Testing for Multiplicative Interactions of Alcohol and Smoking in Multiple Sclerosis

eTable 3. Effect Modification of Alcohol and Smoking Effects by Multiple Sclerosis Diagnosis

eFigure. Directed Acyclic Graph (DAG) Visualizing the Assumed Associations Among the Exposures, Outcomes and Potential Confounders in This Study

This supplementary material has been provided by the authors to give readers additional information about their work.

© 2022 Kleerekooper I et al. *JAMA Network Open*.

eTable 1. Univariable and Multivariable Logistic Regression—Odds of Multiple Sclerosis Case Status

		Univariable logistic regression						Multivariable logistic regression					
		Healthy control group compared with multiple sclerosis			Comorbid control group compared with multiple sclerosis			Healthy control group compared with multiple sclerosis			Comorbid control group compared with multiple sclerosis		
		OR	95% CI	p-value	OR	95% CI	p-value	aOR	95% CI	p-value	aOR	95% CI	p-value
**Smoking status**	Never	NA	NA	NA	NA	NA	NA	NA	NA	NA	NA	NA	NA
	Previous	1.51	1.08 – 2.10	**0.01**	1.16	0.83 – 1.61	0.37	1.59	1.12 – 2.25	**0.009**	1.25	0.88 – 1.77	0.20
	Current	2.89	1.90 – 4.30	**<0.001**	2.34	1.54 – 3.48	**<0.001**	3.05	1.95 – 4.64	**<0.001**	2.30	1.48 – 3.51	**<0.001**
**Alcohol consumption**	Low	NA	NA	NA	NA	NA	NA	NA	NA	NA	NA	NA	NA
	Moderate	0.51	0.36 – 0.73	**<0.001**	0.68	0.48 – 0.97	**0.03**	0.62	0.43 – 0.91	**0.01**	0.81	0.56 – 1.18	0.26
	High	0.63	0.41 – 0.96	**0.03**	0.90	0.59 – 1.37	0.63	0.76	0.48 – 1.20	0.24	1.11	0.70 – 1.75	0.65
**Sex**	Male	NA	NA	NA	NA	NA	NA	NA	NA	NA	NA	NA	NA
	Female	2.64	1.92 – 3.70	**<0.001**	2.16	1.60 – 3.03	**<0.001**	2.97	2.10 – 4.27	**<0.001**	2.28	1.62 – 3.28	**<0.001**
**Age group**	40-49 years	NA	NA	NA	NA	NA	NA	NA	NA	NA	NA	NA	NA
	50-59 years	1.18	0.82 – 1.74	0.38	1.12	0.77 – 1.64	0.56	1.08	0.74 – 1.61	0.69	1.17	0.79 – 1.73	0.44
	60-70 years	1.08	0.74 – 1.61	0.68	0.62	0.42 – 0.92	**0.02**	0.98	0.65 – 1.49	0.94	0.67	0.44 – 1.01	0.05
**Townsend deprivation index**	Lowest quartile	NA	NA	NA	NA	NA	NA	NA	NA	NA	NA	NA	NA
	Low-middle quartile	1.47	0.94 – 2.31	0.10	1.37	0.88 – 2.16	0.17	1.40	0.88 – 2.25	0.16	1.32	0.83 – 2.11	0.24
	High-middle quartile	1.35	0.86 – 2.14	0.20	1.29	0.82 – 2.04	0.27	1.21	0.75 – 1.96	0.43	1.17	0.73 – 1.89	0.51
	Highest quartile	1.90	1.24 – 2.94	**0.004**	1.67	1.10 – 2.60	**0.02**	1.57	1.00 – 2.51	0.05	1.41	0.90 – 2.25	0.14
**BMI**	Low/healthy	NA	NA	NA	NA	NA	NA	NA	NA	NA	NA	NA	NA
	Overweight	1.02	0.72 – 1.46	0.89	0.86	0.61 – 1.22	0.39	1.19	0.83 – 1.71	0.34	0.99	0.69 – 1.41	0.94
	Obese	1.56	1.05 – 2.31	**0.03**	0.92	0.62 – 1.36	0.68	1.72	1.15 – 2.56	**0.008**	1.02	0.68 – 1.51	0.93

© 2022 Kleerekooper I et al. *JAMA Network Open*.

**Household passive smoking**	No	NA	NA	NA	NA	NA	NA	NA	NA	NA	NA	NA	NA
	Yes	1.46	0.90 – 2.27	0.11	1.37	0.84 – 2.13	0.18	NA	NA	NA	NA	NA	NA

OR = odds ratio. BMI = body mass index. aOR = adjusted odds ratio. BMI = body mass index. NA = not applicable. Alcohol intake frequency: never or special occasions only (low), drinking once per month up to 4 times per week (moderate) or daily or almost daily (high).

eTable 2. Testing for multiplicative effects of Alcohol and Smoking in multiple sclerosis

		**MS cohort**			
		**mGCIPL change**	**p-value**	**Trend p-value**	**Observations (n)**
**Smoking status**	**Never**	NA	NA	NA	
	**Previous**	-1.68	0.41		
	**Current**	-2.26	0.28		
**Alcohol consumption**	**Low**	NA	NA	NA	
	**Moderate**	-1.32	0.43		
	**High**	-4.21	**0.02**		
**Interaction terms**	**Previous smoking + Moderate alcohol**	1.91	0.45	NA	
	**Previous smoking + High alcohol**	2.27	0.43		
	**Current smoking + Moderate alcohol**	2.54	0.36		
	**Current smoking + High alcohol**	1.65	0.56		176

Alcohol intake frequency: never or special occasions only (low), drinking once per month up to 4 times per week (moderate) or daily or almost daily (high). MS = multiple sclerosis. mGCIPL = macular ganglion cell and inner plexiform layer. NA = not applicable.

© 2022 Kleerekooper I et al. *JAMA Network Open*.

eTable 3. Effect Modification of Alcohol and Smoking Effects by Multiple Sclerosis Diagnosis				
		**mGCIPL µm change**	**p-value**	**Observations**
**Model 1: interaction smoking with multiple sclerosis (adjusted for alcohol intake)**				
**Controls**				
**Smoking status**	Never	NA	NA	
	Previous	-0.04	0.437	
	Current	0.89	**<0.001**	
**Multiple sclerosis cases**				
**Smoking status**	Never	-4.00	**<0.001**	
	Previous	-0.29	0.784	
	Current	-2.14	*0.077*	72,101
**Model 2: interaction alcohol use with multiple sclerosis (adjusted for smoking)**				
**Controls**				
**Alcohol consumption**	Low	NA	NA	
	Moderate	-0.33	**<0.001**	
	High	-0.93	**<0.001**	
**Multiple sclerosis cases**				
**Alcohol status**	Low	-3.99	**<0.001**	
	Moderate	0.03	0.979	
	High	-2.27	*0.074*	72,101

Alcohol intake frequency: never or special occasions only (low), drinking once per month up to 4 times per week (moderate) or daily or almost daily (high). mGCIPL = macular ganglion cell and inner plexiform layer. NA = not applicable.

© 2022 Kleerekooper I et al. *JAMA Network Open*.

eFigure. Directed Acyclic Graph (DAG) Visualizing the Assumed Associations Among the Exposures, Outcomes and Potential Confounders in This Study

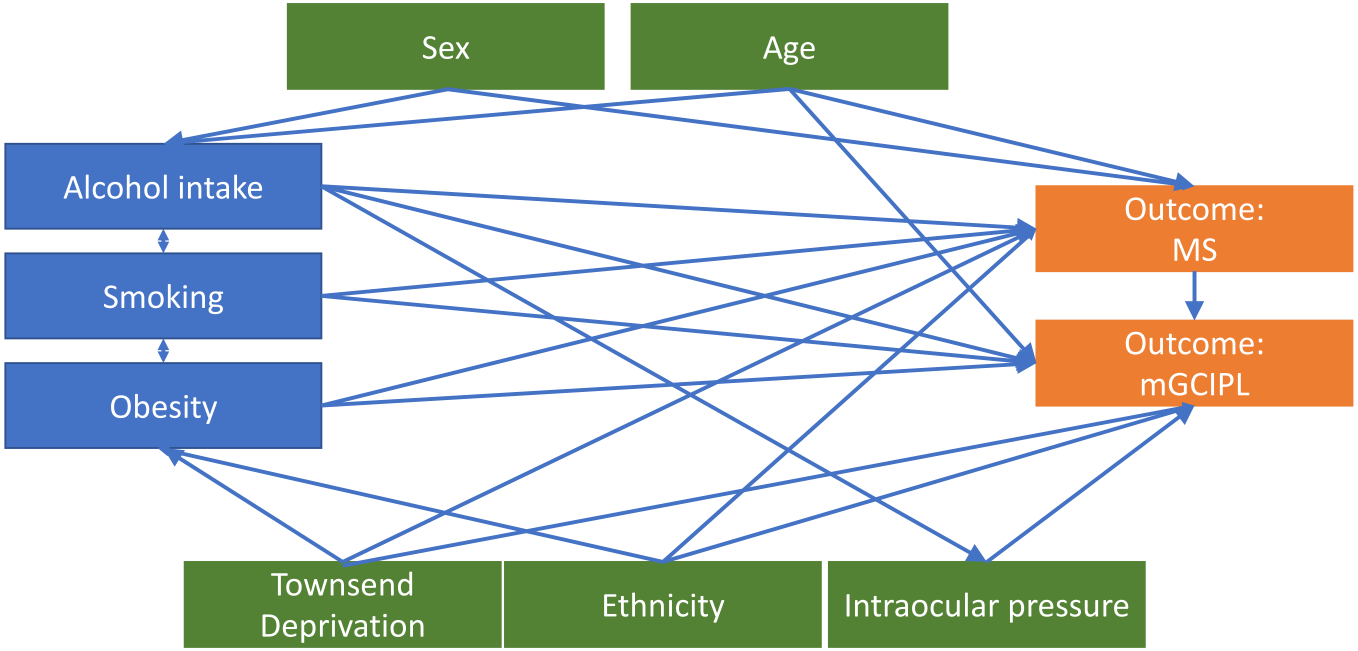

MS = multiple sclerosis. mGCIPL = macular ganglion cell and inner plexiform layer.

© 2022 Kleerekooper I et al. *JAMA Network Open*.
