# Supplementary material for: Associations of Alcohol Consumption and Smoking With Disease Risk and Neurodegeneration in Individuals With Multiple Sclerosis in the United Kingdom
Source: JAMA Netw Open. 2022 Mar 3;5(3):e220902. doi: 10.1001/jamanetworkopen.2022.0902 (PMC8895260; doi:10.1001/jamanetworkopen.2022.0902)
Supplement: Supplement 2. — Nonauthor Collaborators [file jamanetwopen-e220902-s002.pdf]

Supplemental Online Content: Nonauthor Collaborators

*Indicates required information. Only first name, last name, and suffix will appear in PubMed.

*Group Name(s): UK Biobank Eye and Vision Consortium							
***First Name and Middle Initial(s)**	***Last Name**	***Suffix (eg, Jr, III)**	Academic Degrees	Institution	Location (city, state/province, country)	Role or Contribution, eg, chair, principal investigator	Group (if more than 1 Group listed in the byline) and/or Subgroup (eg, Steering Committee)
Naomi	Allen		BSc, MSc, DPhil	University of Oxford	Oxford, England, United Kingdom	principal investigator	
Tariq	Aslam		MA, MB BCh, DPhil, MD	The University of Manchester	Manchester, England, United Kingdom	principal investigator	
Denize	Atan		B.M,B.Ch(Oxon), MA(Cantab), PhD	University of Bristol	Bristol, England, United Kingdom		
Sarah	Barman		PhD	Kingston University	London, England, United Kingdom	principal investigator	
Jenny	Barrett		BA, MSc, PhD	University of Leeds	Leeds, West Yorkshire, United Kingdom	principal investigator	
Paul	Bishop		B Med Sci (Hons), BM BS, PhD	The University of Manchester	Manchester, England, United Kingdom	principal investigator	
Graeme	Black		MA, MB BCh, DPhil	The University of Manchester	Manchester, England, United Kingdom	principal investigator	
Catey	Bunce		BSc (Hons), MSc, DSc	King’s College London	London, England, United Kingdom		
Roxana	Carare		MD, PhD	University of Southampton	Southampton, England, United Kingdom	principal investigator	

*Indicates required information. Only first name, last name, and suffix will appear in PubMed.

***First Name and Middle Initial(s)**	***Last Name**	***Suffix (eg, Jr, III)**	Academic Degrees	Institution	Location (city, state/province, country)	Role or Contribution, eg, chair, principal investigator	Group (if more than 1 Group listed in the byline) and/or Subgroup (eg, Steering Committee)
Usha	Chakravarthy		MD, PhD	Queen's University Belfast	Belfast, Northern Ireland, United Kingdom	principal investigator	
Michelle	Chan		BSc, MBBS	Moorfields Eye Hospital	London, England, United Kingdom		
Sharon	Chua		B.Optom, PhD	UCL Institute of Ophthalmology	London, England, United Kingdom	research support	
Valentina	Cipriani		BSc, PhD	UCL Institute of Ophthalmology	London, England, United Kingdom		
Alexander	Day		MBBS, BSc, PhD	Moorfields Eye Hospital	London, England, United Kingdom	principal investigator	
Parul	Desai		PBM ChB PhD FRCOphth	Moorfields Eye Hospital	London, England, United Kingdom		
Bal	Dhillon		BMedSci, BMBS	University of Edinburgh	Edinburgh, Scotland, United Kingdom	principal investigator	
Andrew	Dick		B.Sc., M.B.B.S.(Lond.), M.D.(Aberd.),	University of Bristol	Bristol, England, United Kingdom	co-chair	
Alexander	Doney		PhD	University of Dundee	Dundee, Scotland, United Kingdom		
Cathy	Egan		MBBS	Moorfields Eye Hospital	London, England, United Kingdom		
Sarah	Ennis		BSc, PhD	University of Southampton	Southampton, England, United Kingdom	principal investigator	

Supplemental Online Content: Nonauthor Collaborators

*Indicates required information. Only first name, last name, and suffix will appear in PubMed.

***First Name and Middle Initial(s)**	***Last Name**	***Suffix (eg, Jr, III)**	Academic Degrees	Institution	Location (city, state/province, country)	Role or Contribution, eg, chair, principal investigator	Group (if more than 1 Group listed in the byline) and/or Subgroup (eg, Steering Committee)
Paul	Foster		BMedSci(Hons), BM, BS, PhD	UCL Institute of Ophthalmology	London, England, United Kingdom	principal investigator	
Marcus	Fruttiger		BSc, PhD	UCL Institute of Ophthalmology	London, England, United Kingdom	principal investigator	
John	Gallacher		BSc, PhD	University of Oxford	Oxford, England, United Kingdom	principal investigator	
David (Ted)	Garway-Heath		MB ChB	UCL Institute of Ophthalmology	London, England, United Kingdom	principal investigator	
Jane	Gibson		BSc(Hons), MSc, PhD	University of Southampton	Southampton, England, United Kingdom		
Dan	Gore		MBBS	Moorfields Eye Hospital	London, England, United Kingdom		
Jeremy	Guggenheim		BSc, PhD	Cardiff University	Cardiff, Wales, United Kingdom	principal investigator	
Chris	Hammond		MA(Cantab), MB ChB, MD	King's College London	London, England, United Kingdom	principal investigator	
Alison	Hardcastle		BSc(Hons), PhD	UCL Institute of Ophthalmology	London, England, United Kingdom	principal investigator	
Simon	Harding		MB ChB, MD	University of Liverpool	Liverpool, England, United Kingdom	principal investigator	
Ruth	Hogg		PhD	Queen's University Belfast	Belfast, Northern Ireland, United Kingdom		
Pirro	Hysi		MD, DPhil	King's College London	London, England, United Kingdom	principal investigator	
Pearse	Keane		BMedSci, MSc, MD	UCL Institute of Ophthalmology	London, England, United Kingdom		

*Indicates required information. Only first name, last name, and suffix will appear in PubMed.

***First Name and Middle Initial(s)**	***Last Name**	***Suffix (eg, Jr, III)**	Academic Degrees	Institution	Location (city, state/province, country)	Role or Contribution, eg, chair, principal investigator	Group (if more than 1 Group listed in the byline) and/or Subgroup (eg, Steering Committee)
Sir Peng Tee	Khaw		MB, PhD	UCL Institute of Ophthalmology	London, England, United Kingdom	principal investigator	
Anthony	Khawaja		MA(Cantab), MPhil, PhD	Moorfields Eye Hospital	London, England, United Kingdom	principal investigator	
Gerassimos	Lascaratos		MBBS, MSc, PhD	Moorfields Eye Hospital	London, England, United Kingdom		
Thomas	Littlejohns		BSc, MSc, PhD	University of Oxford	Oxford, England, United Kingdom		
Andrew	Lotery		MB BCh BAO, MD	University of Southampton	Southampton, England, United Kingdom	chair	
Phil	Luthert		MBBS, PhD	UCL Institute of Ophthalmology	London, England, United Kingdom	principal investigator	
Tom	Macgillivray		BSc, MSc, PhD	University of Edinburgh	Edinburgh, Scotland, United Kingdom		
Sarah	Mackie		BA, BM BCh, PhD	University of Leeds	Leeds, West Yorkshire, United Kingdom		
Bernadette	McGuinness		MBBS, MD, PhD	Queen's University Belfast	Belfast, Northern Ireland, United Kingdom	principal investigator	
Gareth	McKay		BSc, PhD	Queen's University Belfast	Belfast, Northern Ireland, United Kingdom		
Martin	McKibbin		MBBS	Leeds Teaching Hospitals NHS Trust	Leeds, West Yorkshire, United Kingdom	principal investigator	
Danny	Mitry		MB, PhD	University of Edinburgh	Edinburgh, Scotland, United Kingdom	principal investigator	

*Indicates required information. Only first name, last name, and suffix will appear in PubMed.

***First Name and Middle Initial(s)**	***Last Name**	***Suffix (eg, Jr, III)**	Academic Degrees	Institution	Location (city, state/province, country)	Role or Contribution, eg, chair, principal investigator	Group (if more than 1 Group listed in the byline) and/or Subgroup (eg, Steering Committee)
Tony	Moore		BM ChB FRCOphth MD	UCL Institute of Ophthalmology	London, England, United Kingdom	principal investigator	
James	Morgan		MA, DPhil, BM BCH	Cardiff University	Cardiff, Wales, United Kingdom	principal investigator	
Zaynah	Muthy		BSc, MSc	UCL Institute of Ophthalmology	London, England, United Kingdom	research support	
Eoin	O'Sullivan		MB ChB?	King's College Hospital	London, England, United Kingdom		
Chris	Owen		PhD	St George's, University of London	London, England, United Kingdom	principal investigator	
Praveen	Patel		MBBS, MA, MD(Res)	Moorfields Eye Hospital	London, England, United Kingdom	principal investigator	
Euan	Paterson		BSc(Hon), PhD	Queen's University Belfast	Belfast, Northern Ireland, United Kingdom		
Tunde	Peto		PhD	Queen's University Belfast	Belfast, Northern Ireland, United Kingdom	principal investigator	
Axel	Petzold		MD, PhD	UCL Institute of Neurology	London, England, United Kingdom		
Nikolas	Pontikos		BSc, M.Eng (hons), PhD	UCL Institute of Ophthalmology	London, England, United Kingdom		
Jugnoo	Rahi		MBBS, MSc, PhD	UCL Institute of Child Health	London, England, United Kingdom	principal investigator	
Alicja	Rudnicka		BSc, MSc, PhD?	St George's, University of London	London, England, United Kingdom	principal investigator	

*Indicates required information. Only first name, last name, and suffix will appear in PubMed.

***First Name and Middle Initial(s)**	***Last Name**	***Suffix (eg, Jr, III)**	Academic Degrees	Institution	Location (city, state/province, country)	Role or Contribution, eg, chair, principal investigator	Group (if more than 1 Group listed in the byline) and/or Subgroup (eg, Steering Committee)
Jay	Self		BM, PhD	University of Southampton	Southampton, England, United Kingdom		
Panagiotis	Sergouniotis		MBBS, MD, PhD	The University of Manchester	Manchester, England, United Kingdom		
Sobha	Sivaprasad		MS Ophth, DM	Moorfields Eye Hospital	London, England, United Kingdom	principal investigator	
David	Steel		MB ChB, MD	Newcastle University	Newcastle, England, United Kingdom		
Irene	Stratton		BSc, MSc	Gloucestershire Hospitals NHS Foundation Trust	Gloucester, England, United Kingdom		
Nicholas	Strouthidis		MBBS, MD, PhD	Moorfields Eye Hospital	London, England, United Kingdom		
Cathie	Sudlow		MB, MSc, DPhil	University of Edinburgh	Edinburgh, Scotland, United Kingdom	principal investigator	
Zihan	Sun		MBBS, MMed, PhD	UCL Institute of Ophthalmology	London, England, United Kingdom	research support	
Robyn	Tapp		BA, PhD	St George's, University of London	London, England, United Kingdom	principal investigator	
Caroline	Thaung		MBBS, DPhil	UCL Institute of Ophthalmology	London, England, United Kingdom		
Dhanes	Thomas		MB ChB, MD	Moorfields Eye Hospital	London, England, United Kingdom		
Emanuele	Trucco		MSc, PhD	University of Dundee	Dundee, Scotland, United Kingdom		
Adnan	Tufail		MBBS, MD	Moorfields Eye Hospital	London, England, United Kingdom	principal investigator	
Stephen	Vernon		MBBS, MD	Nottingham University Hospitals NHS Trust	Nottingham, England, United Kingdom	principal investigator	

Supplemental Online Content: Nonauthor Collaborators

*Indicates required information. Only first name, last name, and suffix will appear in PubMed.

***First Name and Middle Initial(s)**	***Last Name**	***Suffix (eg, Jr, III)**	Academic Degrees	Institution	Location (city, state/province, country)	Role or Contribution, eg, chair, principal investigator	Group (if more than 1 Group listed in the byline) and/or Subgroup (eg, Steering Committee)
Ananth	Viswanathan		BSc(Hons), MD, PhD	Moorfields Eye Hospital	London, England, United Kingdom	principal investigator	
Veronique	Vitart		Msc, PhD	University of Edinburgh	Edinburgh, Scotland, United Kingdom		
Katie	Williams		MBChB, PhD	King's College London	London, England, United Kingdom		
Cathy	Williams		B.Sc., MBBS, Ph.D	University of Bristol	Bristol, England, United Kingdom	principal investigator	
Jayne	Woodside		PhD	Queen's University Belfast	Belfast, Northern Ireland, United Kingdom	principal investigator	
Max	Yates		BSc (Hons), MBBS, MSc, PhD	University of East Anglia	Norwich, England, United Kingdom	principal investigator	
Jennifer	Yip		BM BS PhD	University of Cambridge	Cambridge, England, United Kingdom	research support	
Yalin	Zheng		PhD	University of Liverpool	Liverpool, England, United Kingdom		
